# Precision detection of recent HIV infections using high-throughput genomic incidence assay

**DOI:** 10.1128/spectrum.02285-23

**Published:** 2023-09-15

**Authors:** Gina Faraci, Sung Yong Park, Tanzy M. T. Love, Michael P. Dubé, Ha Youn Lee

**Affiliations:** 1 Department of Molecular Microbiology and Immunology, Keck School of Medicine, University of Southern California, Los Angeles, California, USA; 2 Department of Biostatistics and Computational Biology, School of Medicine and Dentistry, University of Rochester, Rochester, New York, USA; 3 Division of Infectious Diseases, Department of Medicine, Keck School of Medicine, University of Southern California, Los Angeles, California, USA; National Chung Hsing University, Taichung, Taiwan

**Keywords:** HIV incidence, next-generation sequencing, genomic epidemiology, genomic surveillance

## Abstract

HIV incidence is a key measure for tracking disease spread and identifying populations and geographic regions where new infections are most concentrated. The HIV sequence population provides a robust signal for the stage of infection. Large-scale and high-precision HIV sequencing is crucial for effective genomic incidence surveillance. We produced 1,034 full-length envelope gene sequences from a seroconversion cohort by conducting HIV microdrop sequencing and measuring the genomic incidence assay’s genome similarity index (GSI) dynamics. The measured dynamics of 9 of 12 individuals aligned with the GSI distribution estimated independently using 417 publicly available incident samples. We enhanced the capacity to identify individuals with recent infections, achieving predicted detection accuracies of 92% (89%–94%) for cases within 6 months and 81% (74%–87%) for cases within 9 months. These accuracy levels agreed with the observed detection accuracy intervals of an independent validation data set. Additionally, we produced 131 full-length envelope gene sequences from eight individuals with chronic HIV infection. This analysis confirmed a false recency rate (FRR) of 0%, which was consistent with 162 publicly available chronic samples. The mean duration of recent infection (MDRI) was 238 (209–267) days, indicating an 83% improvement in performance compared to current recent infection testing algorithms. The shifted Poisson mixture model was then used to estimate the time since infection, and the model estimates showed an 88% consistency with the days post infection derived from HIV RNA test dates and/or seroconversion dates. HIV microdrop sequencing provides unique prospects for large-scale incidence surveillance using high-throughput sequencing.

**Importance:**

Accurate identification of recently infected individuals is vital for prioritizing specific populations for interventions, reducing onward transmission risks, and optimizing public health services. However, current HIV-specific antibody-based methods have not been satisfactory in accurately identifying incident cases, hindering the use of HIV recency testing for prevention efforts and partner protection. Genomic incidence assays offer a promising alternative for identifying recent infections. In our study, we used microdroplet technologies to produce a large number of complete HIV envelope gene sequences, enabling the accurate detection of early infection signs. We assessed the dynamics of the incidence assay’s metrics and compared them with statistical models. Our approach demonstrated high accuracy in identifying individuals with recent infections, achieving predicted detection rates exceeding 90% within 6 months and over 80% within 9 months of infection. This high-resolution method holds significant potential for enhancing the effectiveness of HIV incidence screening for case-based surveillance in public health initiatives.

## INTRODUCTION

Accurate measurement of HIV incidence is integral to assessing the HIV/AIDS epidemic over time and across geographic regions and evaluating intervention and prevention programs (1 – 5). Identifying newly infected individuals can help prioritize the target population for interventions, reducing the risk of onward transmissions and maximizing the impact of public health services (6).

The recent infection testing algorithm (RITA) has been used for incidence estimation in a range of cross-sectional surveys, including Population HIV Impact Assessment (PHIA) surveys and the South Africa National HIV Prevalence, Incidence, Behavior and Communication Surveys (7 – 11). However, one of the key performance metrics of HIV incidence assays, the mean duration of recent infection (MDRI) was estimated to be 130 days for RITA, indicating that around 64% of incident cases cannot be identified (12). Additionally, the MDRI of RITA is dependent on the subtype of HIV, which requires local adjustment of MDRI based on the subtype distribution in the region (13). The high likelihood of recently infected individuals being misclassified can impede the use of HIV recency testing for case-based surveillance and partner protection efforts (14).

The genomic incidence assay has offered greater precision in determining the stage of infection compared to current serologic approaches (15). The genomic incidence assay detects the similarity of HIV envelope gene sequences as a signature of recent infection (16 – 19). To effectively determine genomic variability for population incidence surveillance, it is necessary to implement high-throughput sequencing technologies to process a large volume of samples while minimizing sequencing errors. HIV microdrop sequencing has addressed these challenges by labeling HIV templates with a unique molecular identifier (UMI) (20 – 22), amplifying them in around 20,000 microreactors (15), and sequencing the full-length HIV envelope gene by long-read high-throughput sequencing that produces millions of reads with greater than 99.9% accuracy (23).

In this study, we used HIV microdrop sequencing to investigate the incidence assay’s genome similarity index (GSI) dynamics from the study participants, who were serially followed from their early stages of infection in the Centers for Disease Control and Prevention (CDC) seroconversion cohort. The GSI dynamics were quantitatively evaluated via statistical modeling of population-wide variability. We then calculated the MDRI by choosing a threshold with the false recency rate (FRR) at 0% to eliminate the uncertainty associated with FRR. From the full-length HIV envelope gene sequences, we estimated the timing of infection using an acute HIV evolution model. We also evaluated the assay’s performance with chronic cases from the Los Angeles County-University of Southern California (LAC-USC) Rand Schrader clinic cohort.

## MATERIALS AND METHODS

### Study cohorts

We sequenced 62 specimens collected from 12 study participants who were serially followed in the CDC seroconversion cohort from the CASPIR [CDC & Agency for Toxic Substances and Disease Registry (ATSDR) Specimen Packaging, Inventory and Repository] (CDC IRB protocol #4660) (24). Both HIV RNA negative and positive test dates were available (Table 1), allowing us to determine the interval of infection time. Furthermore, enzyme immunoassay (EIA) test records (Table 1) provided us with each individual’s seroconversion timeframe. The samples collected before seroconversion were at Fiebig stage I or II, and their time since infection was estimated as 19.5 (13–34) days (25, 26). Infection time estimates are listed in Table 2 (see the Supplementary Material for more details).

**TABLE 1:** HIV RNA test dates and EIA test dates of the CDC seroconversion cohort

Study participant	HIV RNA last negative date	HIV RNA first positive date	HIV EIA last negative date	HIV EIA first positive date
SC4	3/14/2008	11/17/2008	11/17/2008	1/14/2009
SC5	9/30/2008	12/16/2008	9/30/2008	12/16/2008
SC8	8/1/2008	8/27/2008	8/27/2008	9/26/2008
SC15	3/12/2009	4/3/2009	4/3/2009	5/13/2009
SC18	12/16/2008	4/2/2009	4/2/2009	5/2/2009
SC19	Not available	7/28/2009	7/28/2009	8/27/2009
SC20	Not available	8/3/2009	8/3/2009	8/24/2009
SC21	3/10/2008	11/9/2009	3/10/2008	11/9/2009
SC22	Not available	10/30/2009	10/30/2009	11/10/2009
SC23	Not available	9/4/2009	9/4/2009	10/30/2009
SC24	Not available	1/8/2010	1/8/2010	1/29/2010
SC25	Not available	1/21/2010	1/21/2010	2/1/2010

**TABLE 2:** Serial specimens from CDC seroconversion cohort^*a*^

Specimens	Sample collection date	Days post infection from HIV RNA test	Estimated days post infection	Number of envelope gene sequences
SC4-1	12/8/2008	(21–269)	40.5 (34–55)	16
SC4-2	1/14/2009	(58–306)	77.5 (71–92)	16
SC4-3	2/11/2009	(86–334)	105.5 (99–120)	14
SC4-4	3/18/2009	(121–369)	140.5 (134–155)	5
SC4-5	4/15/2009	(149–397)	168.5 (162–183)	6
SC4-6	5/14/2009	(178–426)	197.5 (191–212)	16
SC4-7	6/17/2009	(212–460)	231.5 (225–246)	14
SC4-8	11/20/2009	(368–616)	387.5 (381–402)	16
SC4-9	12/16/2009	(394–642)	413.5 (407–428)	6
SC5-1	12/16/2008	(0–77)	38.5 (0–77)^*b*^	9
SC5-4	3/5/2009	(79–156)	117.5 (79–156)	41
SC5-5	4/13/2009	(118–195)	156.5 (118–195)	17
SC5-6	5/12/2009	(147–224)	185.5 (147–224)	19
SC5-8	9/14/2009	(272–349)	310.5 (272–349)	6
SC8-1	1/13/2009	(139–165)	158.5 (152–173)	13
SC8-2	2/19/2009	(176–202)	195.5 (189–210)	18
SC8-3	3/19/2009	(204–230)	223.5 (217–238)	13
SC8-6	6/23/2009	(300–326)	319.5 (313–334)	22
SC8-7	7/21/2009	(328–354)	347.5 (341–362)	7
SC15-1	4/14/2009	(11–33)	30.5 (24–45)	30
SC15-2	5/13/2009	(40–62)	59.5 (53–74)	56
SC15-3	6/17/2009	(75–97)	94.5 (88–109)	62
SC18-1	6/19/2009	(78–185)	97.5 (91–112)	22
SC18-2	7/13/2009	(102–209)	121.5 (115–136)	26
SC18-3	8/7/2009	(127–234)	146.5 (140–161)	23
SC18-4	9/14/2009	(165–272)	184.5 (178–199)	5
SC18-5	10/5/2009	(186–293)	205.5 (199–220)	5
SC19-1	9/17/2009	(51–)	70.5 (64–85)	5
SC19-2	10/1/2009	(65–)	84.5 (78–99)	18
SC19-8	3/15/2010	(230–)	249.5 (243–264)	26
SC19-9	7/12/2010	(349–)	368.5 (362–383)	24
SC20-1	8/24/2009	(21–)	40.5 (34–55)	17
SC20-2	10/2/2009	(60–)	79.5 (73–94)	5
SC20-3	10/26/2009	(84–)	103.5 (97–118)	7
SC20-4	12/3/2009	(122–)	141.5 (135–156)	6
SC20-8	4/27/2010	(267–)	286.5 (280–301)	12
SC20-9	6/8/2010	(309–)	328.5 (322–343)	9
SC20-10	7/20/2010	(351–)	370.5 (364–385)	13
SC21-1	11/9/2009	(0–609)	52.2 (34.3–70.2)^*c*^	11 (B/F1(5)]
SC21-3	12/30/2009	(51–660)	103.2 (85.3–121.2)	18 (B/F1(10)]
SC21-4	2/3/2010	(86–695)	138.2 (120.3–156.2)	15 (B/F1(7)]
SC21-5	2/26/2010	(109–718)	161.2 (143.3–179.2)	15 (B/F1(9), B/D (1)]
SC21-6	3/29/2010	(140–749)	192.2 (174.3–210.2)	20 B/F1(13)]
SC22-1	11/10/2009	(11–)	30.5 (24–45)	6
SC22-6	4/5/2010	(157–)	176.5 (170–191)	5
SC23-2	2/4/2010	(153–)	172.5 (166–187)	9
SC23-3	3/8/2010	(185–)	204.5 (198–219)	6
SC23-7	7/6/2010	(305–)	324.5 (318–339)	24
SC23-8	7/22/2010	(321–)	340.5 (334–355)	20
SC24-2	1/29/2010	(21–)	40.5 (34–55)	21
SC24-3	3/10/2010	(61–)	80.5 (74–95)	26
SC24-4	4/7/2010	(89–)	108.5 (102–123)	19
SC24-6	6/8/2010	(151–)	170.5 (164–185)	26
SC24-7	7/6/2010	(179–)	198.5 (192–213)	15
SC24-8	7/22/2010	(195–)	214.5 (208–229)	11
SC24-9	8/4/2010	(208–)	227.5 (221–242)	17
SC24-10	8/18/2010	(222–)	241.5 (235–256)	12
SC25-2	3/3/2010	(41–)	60.5 (54–75)	14 [B/D (7)]
SC25-3	4/7/2010	(76–)	95.5 (89–110)	5 [B/D (3)]
SC25-5	6/16/2010	(146–)	165.5 (159–180)	23 [B/D (18)]
SC25-7	7/22/2010	(182–)	201.5 (195–216)	27 [B/D (19)]
SC25-8	8/4/2010	(195–)	214.5 (208–229)	24 [B/D (13)]

^*a*^ Sample collection date, days post infection by RNA test dates (if available), estimated days post infection by seroconversion dates (if available), and the number of full-length envelope gene sequences we obtained. The majority of the sequences were subtype B and recombinant sequences were indicated by the number of sequences in parentheses, as assigned by the REGA HIV-1 subtyping tool (27).

^*b*^ Half-time point of HIV RNA last negative and first positive dates.

^*c*^ Estimated using SPMM.

We also studied eight study participants who were enrolled at the LAC-USC Rand Schrader Clinic (USC IRB protocol #HS-12–00121), as previously reported (17). Participants were confirmed to be chronically infected at the time of sample collection, based on their documented first positive HIV antibody test records [enzyme-linked immunosorbent assay (ELISA) and Western blot analysis] or due to an AIDS diagnosis with CD4^+^ T cell count below 200 cells/mm^3^ (Table 3). The study IDs of study participants were anonymized by assigning serial numbers for the CDC seroconversion cohort and random codes for the Rand Schrader Cohort. All study participants provided written informed consent at enrollment.

**TABLE 3:** LAC-USC Rand Schrader clinic cohort^*a*^

Study participant	Sample collection date	Minimum days post infection	ART status	Viral load (RNA copies /mL)	CD4^+^ T cell count (cells/mm^3^)	Number of envelope gene sequences
UD9992^*b*^	7/2/2012	48	Naive	1,230	185	16
CX7332	8/7/2012	5,464	Experienced	389,081	63	14 [B/D (1)]
NK9147^*b*^	8/8/2012	65	Naive	156,582	87	25
EC8287	8/8/2012−9/11/2012	427	Naive	46,398	591	8
CS0442	10/23/2012	1,947	Experienced	1,980	58	14
CD8867	12/20/2012	3,513	Experienced	22,136	284	14
SK4851	1/9/2013	800	Experienced	91,406	208	27
JN1992	6/5/2013	1,250	Not available	7,328	245	13

^*a*^ Sample collection date, minimum days post infection, ART status, viral load, CD4^+^ T cell count, and the number of full-length envelope gene sequences we obtained. Most sequences were subtype B and recombinant sequences were marked with the number of sequences in parenthesis.

^*b*^ Study participants who had an AIDS diagnosis at the time of specimen collection with CD4^+^ T cell count less than 200 cells/mm^3^.

### Publicly available incident and chronic specimens

To infer the GSI probability density function, publicly available HIV complete envelope gene sequences from 417 incident specimens were analyzed as previously described (see the Supplementary Material for data sources) (18, 19). An additional 107 publicly available incident samples were used to measure the detection accuracy of recent infections (18, 19). We also analyzed 162 publicly available chronic specimens (18, 19).

### HIV RNA extraction, UMI labeling, and microdroplet amplification

HIV RNA was extracted from study participants’ plasma specimens as previously described (15). The extracted HIV RNA from each specimen was used to synthesize UMI-tagged cDNA with the envelope gene UMI primer, rover1UMIenvB3out (15). UMI-tagged HIV cDNA was then loaded into the QX200 Droplet Generator (BioRad) with PCR mix and PicoSurf-1 oil (Sphere Fluidics) for droplet generation (15). The droplets were then PCR cycled at an annealing temperature of 55°C (15). After purification with Ampure XP beads (Beckman Coulter), the second PCR was performed with 8 µL of purified PCR product at an annealing temperature of 57°C (15).

### Bulk PCR

We additionally amplified UMI-tagged HIV cDNA using conventional bulk PCR methods. Each specimen was amplified in one to four replicate reactions with 2 µL of the UMI-tagged cDNA (15). These were then PCR cycled at an annealing temperature of 55°C. After purification, 2 µL of purified PCR product was subjected to the second round of PCR at an annealing temperature of 57°C.

### Quantification, pooling, and long-read sequencing

The second PCR products from both microdroplet amplification and bulk amplification methods were quantified via the Quant-iT PicoGreen dsDNA Assay (ThermoFisher). Equimolar amounts of each specimen were then pooled, accounting for the presence of untargeted amplicons. The pooled samples were then sequenced using the PacBio Sequel II system (PacBio) at DNA Technologies Core, UC Davis Genome Center (15).

### High-throughput sequencing data analysis

The full-length envelope gene raw sequencing reads were first demultiplexed based on their index sequences. All raw reads sharing the same UMI were then collected and sorted by their lengths. Raw reads with the most frequent length (the length with the highest count in the length histogram) and their closest lengths (when needed) were preferentially selected, with a minimum sequencing depth of 35. These selected sequences were then aligned using MUSCLE (22), and their consensus sequence was obtained. To account for sequencing errors in the UMI region, we removed consensus sequences with low read counts when their UMI had less than three nucleotide base differences (including gaps) from high-read-count consensus sequences. The set of consensus sequences from each specimen was then aligned and trimmed to obtain full-length envelope gene (HXB2 6225–8795) sequences.

### GSI probability density function, MDRI, and detection accuracy

We previously modeled the GSI probability density function as a beta distribution as follows (18), with $\alpha \left(t\right)=V\times \overset{-}{GSI}(t)$ and $\beta \left(t\right)=V\left[1-\overset{-}{GSI}\left(t\right)\right]$ . Here, V is the precision parameter and the average GSI was assumed to decrease as a function of time as follows:


(1) $$\bar{GSI}(t)=c\frac{1+\exp[-M/S]}{1+\exp[(t-M)/S{]}^{\prime }}$$

where *M*, *S,* and *c* are regression parameters. Then, the cumulative density function of GSI at time *t* is given by the regularized incomplete beta function:


(2) $$CDF(GSI|t)=I_{GSI}[\alpha (t),\beta (t)].$$

This model was fitted to the 417 incident samples for which an estimated infection time was available. The parameters were estimated by using the likelihood function (18), under the constraint that the cumulative density function value is 0.014 at GSI = 0.206 and *t* = 22 days. This value was empirically obtained using the subset of 71 incident specimens at Fiebig stage II.

As derived previously (18), the MDRI was obtained from the integral of the regularized incomplete beta function as follows:


(3) $$MDRI=T-\int_{0}^{T}I_{\theta }[\alpha (t),\beta (t)]dt,$$

where T **=** 365 days, and θ is the threshold GSI value that distinguishes chronic from incident infections. The detection accuracy for identifying incident cases within a given time of infection, τ, is the probability that an individual’s GSI value is not below the threshold. This was calculated as


(4) $$\;\;DA\;=\;1-\;\;\frac{1}{\tau }\int_{0}^{\tau }I_{\theta }\left[\alpha \left(t\right),\;\beta \left(t\right)\right]\;dt.$$

## RESULTS

### Incidence assay metric dynamics

**Fig 1:**
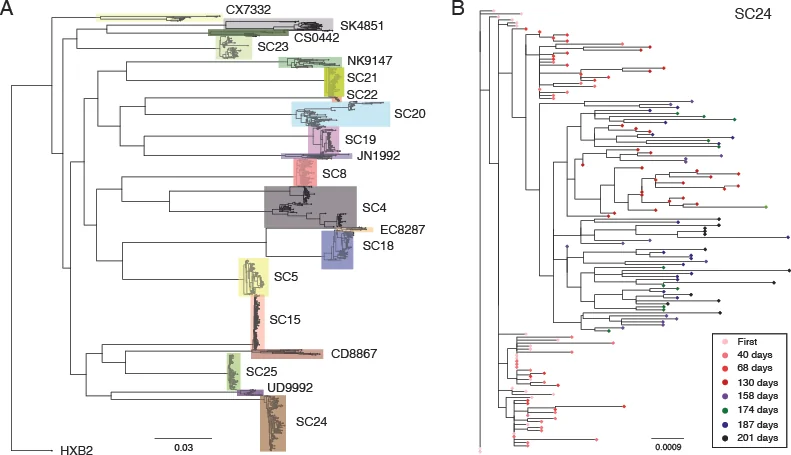
Maximum likelihood trees. (**A**) Maximum likelihood tree of all 1,165 full-length envelope gene sequences from both the CDC seroconversion cohort (denoted by SC) and LAC-USC Rand Schrader Clinic cohort, aligned with the HXB2-envelope sequence. Each colored box represents each study participant’s cluster. The envelope gene sequences were aligned using MAFFT (version 7.392) (28), and the resulting alignment was used to build a phylogenetic tree using FastTree (version 2.1.8) (29). The final tree was visualized using FigTree (version 1.4.4). (**B**) Maximum likelihood tree of 147 full-length envelope gene sequences from study participant SC24 in the CDC seroconversion cohort. The first sample was colored in pink, and subsequent sequences collected at 40, 68, 130, 158, 174, 187, and 201 days after the first sample were colored in light red, red, dark red, purple, green, blue, and black, respectively.

We studied the 62 specimens of 12 ART naive study participants who were serially followed from the early stage of infection in the CDC seroconversion cohort (Tables 1 and 2). We obtained 5–62 full-length envelope gene (consensus) sequences from each specimen using HIV microdrop sequencing (15) and conventional bulk PCR methods, yielding 1,034 full-length envelope gene sequences (Table 2). Additionally, we obtained 131 full-length envelope gene sequences from eight study participants in the LAC-USC Rand Schrader Clinic cohort (Table 3) (17). The phylogenetic tree of all 1,164 envelope gene sequences showed that sequences from the same study participant clustered together (Fig. 1A). The tree of the eight serial samples collected from study participant SC24 demonstrated a pattern of evolutionary divergence increasing over time (Fig. 1B).

**Fig 2:**
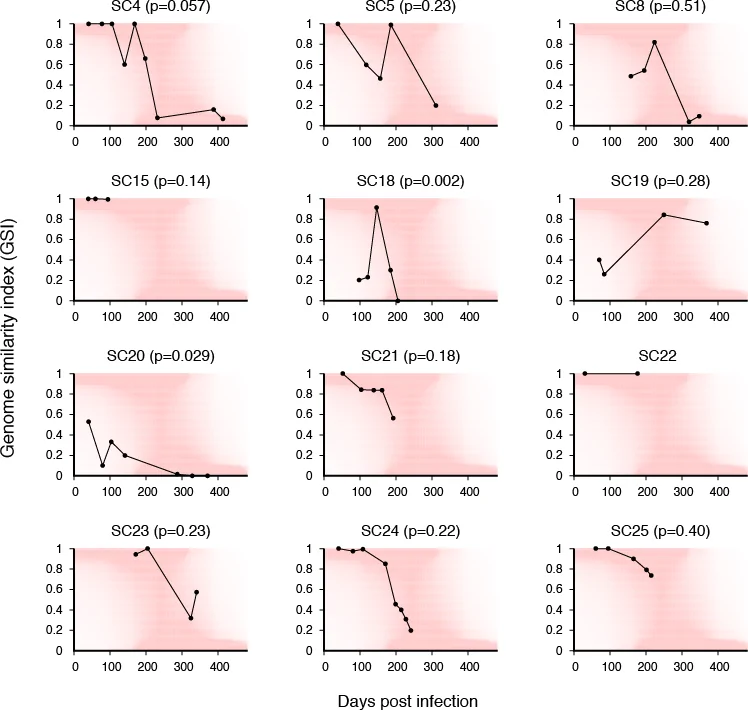
GSI dynamics for 12 individuals’ samples collected at serial visits. Under each individual trajectory, the heatmap (red) showed the fitted densities of the GSI distribution over time. The goodness-of-fit *P*-value was obtained from a one-sample Kolmogorov-Smirnov test.

**Fig 3:**
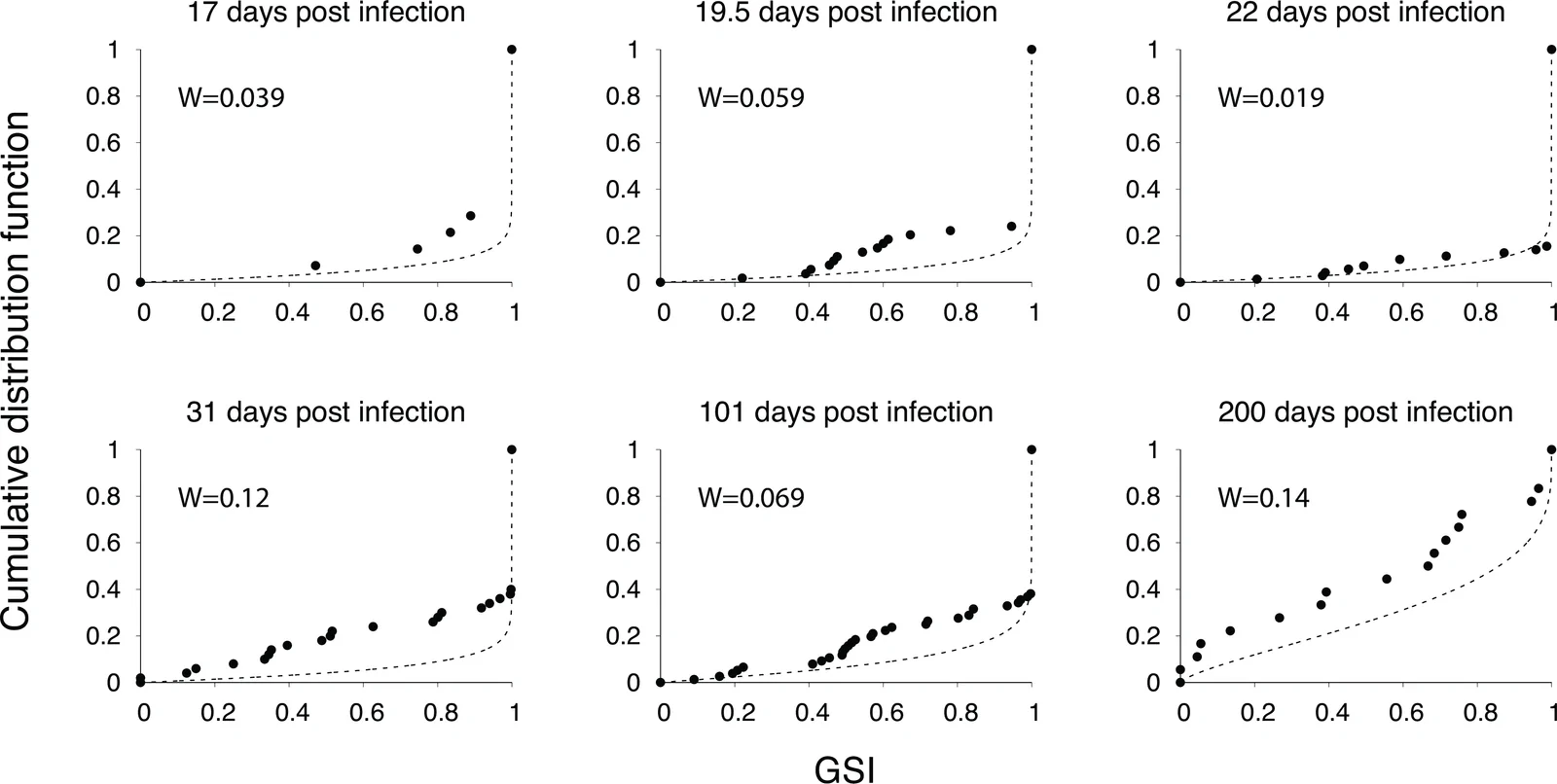
Cumulative distribution functions (CDFs) for GSI. The fitted CDFs for GSI at six values of days post infection (dashed lines), along with the empirical CDFs (points) determined from 14 incident samples collected at 17 days post infection, 54 samples at 19.5 days, 71 samples at 22 days, 50 samples at 31 days, 76 samples at 101 days, and 18 samples collected between 175 and 225 days post infection. The Wasserstein distances, *W*, between the fitted and empirical CDFs are shown in each panel.

We measured the dynamics of the incidence assay’s metric, the GSI, for 12 individuals in the CDC seroconversion cohort. GSI measures the proportion of similar sequences in the viral population of each host, permitting us to properly identify the signature of a recent infection by accounting for multiple founder infections (15, 17, 19). The GSI dynamics were plotted over a heatmap of the fitted density for the GSI distribution over time (Fig. 2), where infection times were estimated from HIV RNA test dates, seroconversion dates, and sample collection intervals. We independently estimated the GSI probability density as a function of time using 417 publicly available incident samples within 1 year of infection (Fig. S1) (18, 19). We verified that 99.2% of these 417 samples were located inside the 99% prediction interval (Fig. S1). The estimated cumulative density function closely matched the empirical cumulative distribution of GSI at various time points, as confirmed by low Wasserstein distances ranging from 0.039 to 0.14 and high Pearson correlation coefficients ranging from 0.96 to 0.98 (Fig. 3).

Study participant SC4 was followed for longer than 1 year, from before their seroconversion (Table 2). We observed a decrease in GSI over time, with the GSI dynamics conforming to the high probability density function region (Fig. 2). We performed a one-sample Kolmogorov-Smirnov test to assess how closely the GSI dynamics of SC4 approximated the fitted probability density function. The *P* value for SC4 (0.057) indicates no significant departure from agreement between the measured dynamics and the model distribution. Similarly, the GSI dynamics of study participants SC5, SC8, and SC15 conformed to the model dynamics (*P* = 0.23, *P* = 0.51, and *P* = 0.14). SC22 showed GSI values of 1 in their two specimens, one collected within 50 days and another from close to 200 days of infection (Fig. 2).

In study participant SC18, the GSI values of two samples collected around 100 days post infection were substantially smaller than the model would predict, followed by an increase to nearly 1 and a subsequent decrease in the later time points. This behavior was a significant deviation from the model distribution (*P* = 0.002, Fig. 2). A similar pattern of transient GSI increase was obtained in SC19, but the goodness-of-fit indicated a lack of significant deviation from the model (*P* = 0.28, Fig. 2). SC20 showed an overall decrease in GSI over time. However, this trend significantly deviated from the model distribution, likely due to the relatively low values of GSI at early time points, a signature of multiple founders (*P* = 0.029, Fig. 2 and 5P). Conversely, the GSI dynamics of SC21 conformed to the model prediction when we approximated the time since infection of the first sample as 52.2 days using the shifted Poisson mixture model (SPMM) (*P* = 0.18, Fig. 2 and 5R) (30). The GSI dynamics of SC23, SC24, and SC25 were also consistent with the model prediction (*P* = 0.23, 0.22, and 0.40; Fig. 2).

### Mean duration of recent infection and false recency rate

**Fig 4:**
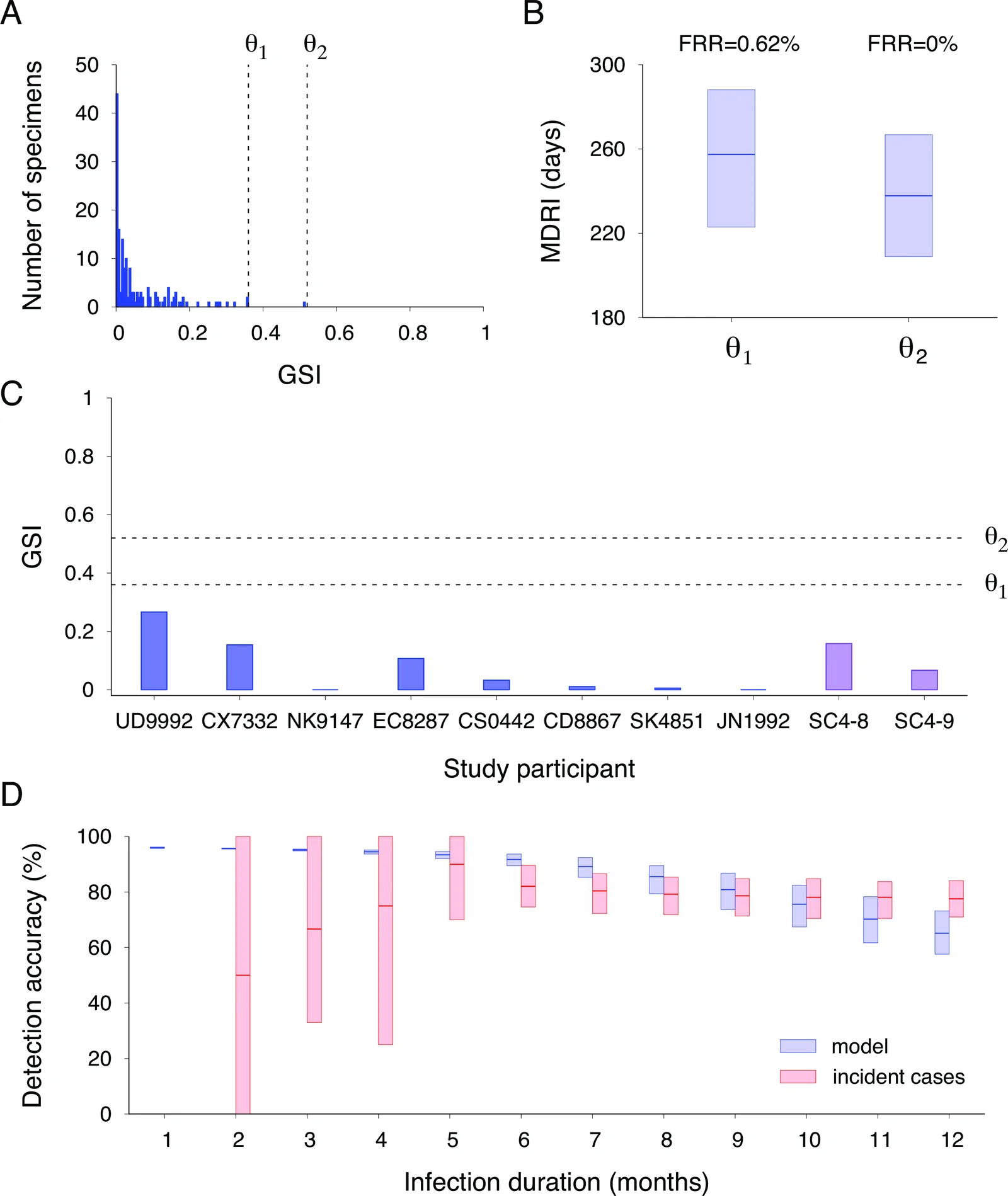
MDRI, FRR, and detection accuracy. (**A**) The GSI distribution of previously published envelope gene sequences from 162 chronic samples with an infection time longer than 1 year (18, 19). Setting a threshold value of 0.36 (denoted as *θ*_1_), the FRR was 0.62% (0%–1.9%). Setting a higher threshold value of 0.52 (denoted as *θ*_2_), the FRR was 0%. (**B**) The MDRI was estimated as 257 (223–288) days for *θ*_1_ and as 238 (209–267) days for *θ*_2_. (**C**) GSI values of eight chronically infected individuals from the LAC-USC Rand Schrader Clinic and two chronic specimens from the CDC seroconversion cohort. These values were below both *θ*_1_ and *θ*_2_ thresholds. (**D**) Detection accuracy of incident cases within a given time of infection using our model (red boxes) and publicly available incident specimens with a maximum infection duration (blue boxes) (18, 19). The model predicted that incident cases within 6 months of infection could be detected with an accuracy of 92% (89%–94%), which overlapped with the observed accuracy of 67 incident cases within 6 months, at 82% (75%–90%). Similarly, the model predicted an accuracy of 81% (74%–87%) for detecting incident cases within 9 months of infection, which overlapped with the measured accuracy for 103 incident cases within 9 months, at 79% (72%–85%).

We investigated the sensitivity of MDRI to varying the GSI cutoff value, θ. Previously published envelope gene sequences from 162 chronic samples, with a documented infection time longer than 1 year, showed GSI values lower than 0.52 (Fig. 4A). Consequently, setting the threshold value to 0.52 yielded an FRR of 0%, which can effectively reduce uncertainty in the incidence rate. Under this condition, the MDRI was estimated to be 238 (209–267) days. This was comparable to the MDRI of 257 (223–288) days when the threshold value was lowered to 0.36 with an FRR = 0.62% (0%–1.9%) (Fig. 4B). The GSI values of an independent set of eight chronically infected individuals of LAC-USC Rand Schrader Clinic and two specimens collected at the chronic stage of the CDC seroconversion cohort were below both threshold values, validating the high specificity of the genomic incidence assay (Fig. 4C).

The detection accuracy of recent cases was assessed as a function of their infection time. As calculated in equation (4), the genomic incidence assay could detect cases within 3 months of infection with 95% (95%–96%) accuracy, cases within 6 months with 92% (89%–94%) accuracy, cases within 9 months with 81% (74%–87%) accuracy, and cases within 1 year with 65% (58%–73%) accuracy (Fig. 4D). To validate our findings, we measured the detection accuracy using 107 publicly available specimens (18, 19), which were 67% (33%–100%) for individuals within 3 months, 82% (75%–90%) for individuals within 6 months, 79% (72%–85%) for individuals within 9 months, and 78% (71%–84%) for individuals within 1 year. These accuracy levels overlapped with the predicted ones every month, as shown in Fig. 4D.

### Estimating time since infection

**Fig 5:**
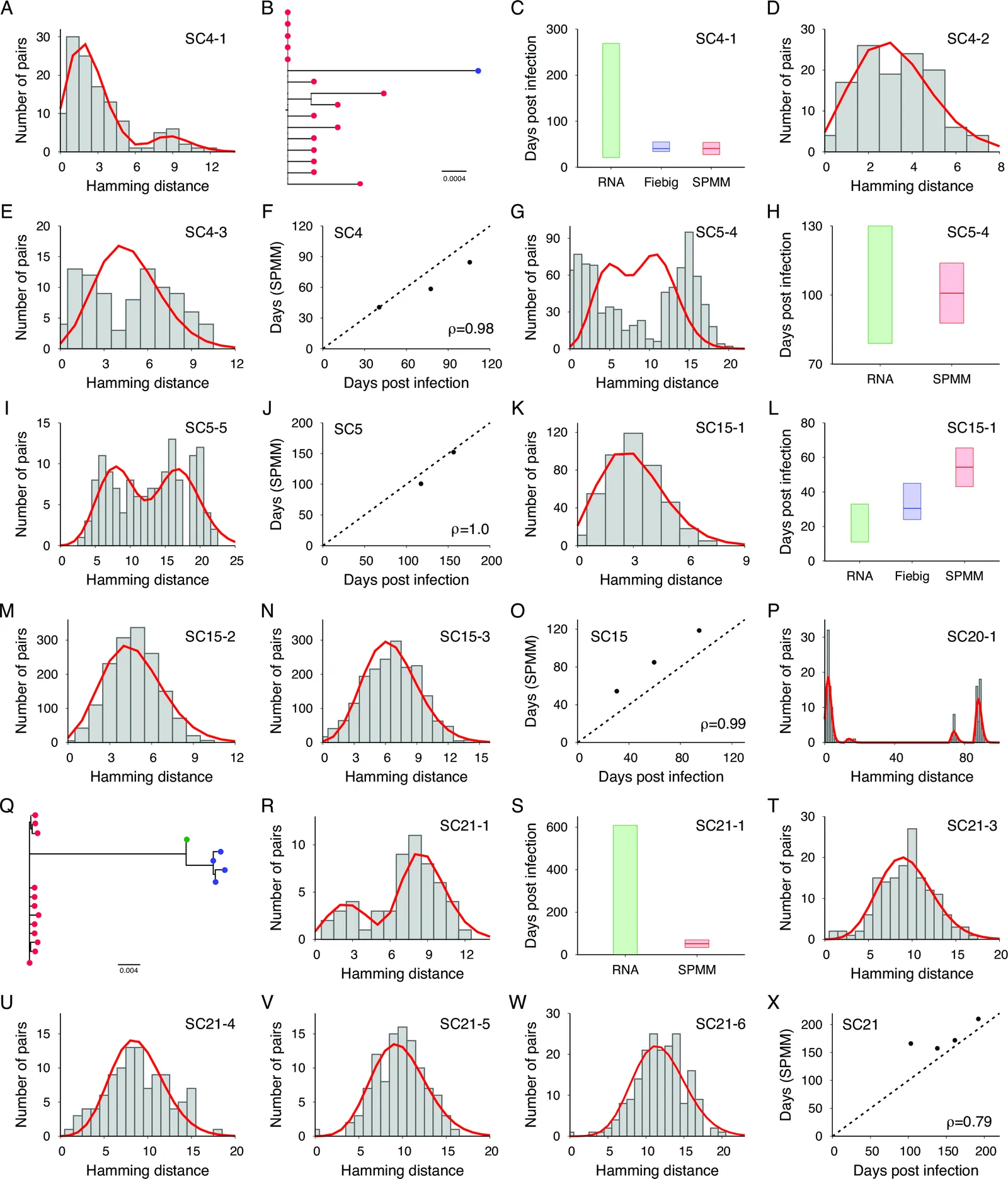
Infection time estimates by SPMM. (**A**) The fit of SPMM (red line) to the Hamming distance distribution of SC4-1’s 16 envelope gene sequences (grey boxes). The number of founder strains was estimated as two and the time since infection was estimated as 40.6 (27.3–53.9). (**B**) Two lineages were colored by red and blue in the phylogenetic tree of SC4-1’s 16 envelope gene sequences. (**C**) Time since infection estimated by SPMM was consistent with HIV RNA test date estimate of (21–269) days and the Fiebig staging estimate of 40.5 (34–55) days. (**D**) The fit of SPMM to the Hamming distance distribution of SC4-2. (**E**) The fit of SPMM to the Hamming distance distribution of SC4-3. (**F**) Our model estimates for the times since infection of the SC4 samples were consistent with the estimates obtained by Fiebig staging and sample collection intervals (Pearson correlation coefficient *Ρ* = 0.98). (**G**) The fit of SPMM to the Hamming distance distribution of SC5-4. (**H**) The model estimate agreed with the infection time range based on dates of the last negative and first positive HIV RNA tests. (**I**) The fit of SPMM to the Hamming distance distribution of SC5-5. (**J**) The model estimates for specimens obtained from SC5 were consistent with the infection times determined by HIV RNA test dates and sample collection intervals (*Ρ* = 1.0). (**K**) The fit of SPMM to the Hamming distance distribution of SC15-1. (**L**) The model estimate for SC15-1 overlapped with the infection time interval determined by Fiebig staging but was greater than the interval determined by the dates of the HIV RNA tests. (**M**) The fit of SPMM to the Hamming distance distribution of SC15-2. (**N**) The fit of SPMM to the Hamming distance distribution of SC15-3. (**O**) SPMM’s infection time estimates were consistent with Fiebig estimates for the SC15’s three samples (*Ρ* = 0.99). (**P**) The SPMM model fit to the Hamming distance distribution of SC20-1’s 17 envelope gene sequences revealed the presence of four peaks, indicating the signature of three founder strains. (**Q**) Three lineages were colored in red, blue, and green in the phylogenetic tree of SC20-1. (**R**) The fit of SPMM to the Hamming distance distribution of SC21-1. (**S**) The model estimate fell within the range determined by the HIV RNA test results. (**T**) The SPMM model fit to the Hamming distance distribution of SC21-3. (**U**) The SPMM model fit to the Hamming distance distribution of SC21-4. (**V**) The SPMM model fit to the Hamming distance distribution of SC21-5. (**W**) The SPMM model fit to the Hamming distance distribution of SC21-6. (**X**) The model estimates were consistent with the sample collection intervals of SC21 (*Ρ* = 0.79).

We estimated time since infection using the SPMM, which quantifies early HIV-1 evolution during acute infections originating from a single or multiple founder viruses (30). Figure 5 shows the Hamming distance distribution of SC4-1’s 16 envelope gene sequences, along with the model fit. The number of founder strains was estimated to be two, as shown in the phylogenetic tree in Fig. 5B. Consistent with the Fiebig staging estimate of 40.5 (34–55) days post infection, the SPMM estimated this sample’s time since infection as 40.6 (27.3–53.9) days (Fig. 5C). The model estimates for subsequent samples were also in agreement with the sample collection intervals (*Ρ* = 0.98, Fig. 5D through F). The model estimates for specimens obtained from SC5 were also consistent with the infection times determined by HIV RNA test dates and sample collection intervals (Fig. 5G through J). The model estimates for SC15’s specimens overlapped with those by Fiebig staging but were greater than those by HIV RNA test dates (Fig. 5K through O).

The Hamming distance distribution of 17 envelope gene sequences obtained from SC20’s first sample revealed a signature of three founder strains, as indicated by four distinct peaks in Fig. 5P. A phylogenetic tree of these envelope gene sequences confirmed the presence of three distinct founders (Fig. 5Q). The infection time of 42.9 (29.7–56.1) days by SPMM matched the Fiebig estimate of 40.5 (34–55). SPMM estimated SC21’s first specimen’s time since infection as 52.2 (34.2–70.2) days (Fig. 5R), which falls within the range determined by HIV RNA test results (Fig. 5S). This participant’s three subsequent samples conformed to SPMM (Fig. 5T through W), resulting in a high level of association between the model estimates and sample collection intervals (*Ρ* = 0.79, Fig. 5X).

The estimated time since infection for SC8-1 using SPMM was higher than the estimates obtained using HIV RNA test date and Fiebig staging (Fig. S2B). The model estimates for SC18’s specimens were not consistent with HIV RNA test date estimates and Fiebig staging, resulting in a negative correlation coefficient (*Ρ*= −0.79, Fig. S2F). On the other hand, SC19, SC24, and SC25 showed a high level of consistency among the SPMM estimates, RNA test date estimates, and Fiebig staging (Fig. S2G through P). Table 4 presents the model fit results for all specimens that had over 10 envelope gene sequences and were collected within 6 months of infection. Out of the 25 specimens analyzed, the 95% CI of infection time estimated by SPMM overlapped with the infection time estimates based on HIV RNA test dates and/or Fiebig staging for 22 specimens (Tables 2 and 4).

**TABLE 4:** Results of shifted Poisson mixture model^*a*^

Specimens	Estimated days post infection	Number of founder viruses	*P*-value	SSE/AIC
SC4-1	40.6 (27.3–53.9)	2	0.73	0.0048/457.8
SC4-2	58.5 (42.5–74.4)	1	0.54	0.0077/467.4
SC4-3	84.6 (64.2–105.0)	1	<0.001	0.051/477.9
SC5-4	100.8 (87.8–113.9)	2	<0.001	0.059/5014.7
SC5-5	152.3 (127.3–177.2)	2	0.034	0.011/713.4
SC8-1	218.2 (183.9–252.5)	1	<0.001	0.017/448.0
SC15-1	54.3 (43.1–65.5)	1	0.027	0.0043/1580.5
SC15-2	84.9 (74.7–95.2)	1	<0.001	0.0047/6257.4
SC15-3	118.5 (107.0–129.9)	1	<0.001	0.0031/9145.5
SC18-1	202.4 (177.2–227.5)	4	<0.001	0.0072/1391.4
SC18-2	231.9 (207.1–256.7)	2	<0.001	0.0022/1921.6
SC18-3	36.5 (26.0–46.9)	2	<0.001	0.0082/976.9
SC19-2	93.0 (74.2–111.7)	6	<0.001	0.0085/755.3
SC20-1	42.9 (29.7–56.1)	3	<0.001	0.023/479.9
SC21-1	52.2 (34.3–70.2)	3	0.48	0.0062/249.5
SC21-3	166.1 (141.1–191.2)	1	<0.001	0.0053/795.4
SC21-4	157.4 (130.7–184.2)	1	<0.001	0.0078/577.0
SC21-5	171.9 (144.0–199.8)	1	<0.001	0.0043/524.7
SC21-6	210.1 (183.4–236.9)	1	<0.001	0.0056/996.1
SC24-2	60.1 (46.1–74.1)	1	<0.001	0.029/769.2
SC24-3	114.7 (97.3–132.1)	1	<0.001	0.0063/1643.0
SC24-4	108.5 (88.7–128.3)	1	0.64	0.0027/788.8
SC24-6	152.1 (132.1–172.1)	1	<0.001	0.0062/1794.8
SC25-2	89.8 (68.7–111.0)	1	<0.001	0.073/510.4
SC25-5	115.5 (96.8–134.2)	1	<0.001	0.053/1716.9

^*a*^ Estimated time since infection, number of founder viruses, goodness-of-fit *P*-value, and sum of squared errors (SSE)/Akaike Information Criteria (AIC).

## DISCUSSION

The primary advantage of our genomic incidence assay is its desirable performance metrics for determining incidence. The genomic approach estimated an FRR of 0% and an MDRI of 238 (209–267) days, representing an 83% increase in performance compared to current recent infection testing algorithms (RITAs) (7 – 11). While our genomic assay can classify 65% (58%–73%) of incident cases as recent overall, it was predicted to have a higher accuracy for identifying individuals who have been more recently infected. The assay would be able to correctly identify 95% (95%–96%) of incidence cases within 3 months of infection, 92% (89%–94%) for cases within 6 months, and 81% (74%–87%) for cases within 9 months. To validate our predictions, we measured the detection accuracy using an independent data set with a maximum duration of infection and found that the accuracy intervals overlapped with the predicted accuracy intervals. The low accuracy of current serologic assays has deterred their use in non-incidence surveillance cases, such as identifying geographic hotspots, infection transmission clusters, and subpopulations with ongoing or emerging transmission (4). Our high-resolution recent infection detection approach provides significant advantages in enhancing targeted prevention efforts and facilitating partner protection programs, thereby maximizing the impact of public health services (14, 31).

Producing over 1,000 full-length HIV envelope gene sequences in a high-throughput setting via HIV microdrop sequencing provided unprecedented opportunities for measuring the dynamics of GSI. Out of 12 participants studied, the GSI dynamics of nine were found to conform to the probability density function of GSI over time, which was independently estimated using 417 incident samples with available infection times. Although GSI identifies recent infection signals by taking multiple founder infections into account, incident samples with multiple founders may still have low GSI values, as observed in study participants SC18, SC19, and SC20. Therefore, caution should be exercised as GSI distributions in populations with a high prevalence of men who have sex with men (MSM) and injection drug users (IDU) may potentially deviate from our model estimates, despite being included in the 417 incidence samples, as these groups have a high likelihood of multiple founder infections (32 – 35).

We used the shifted Poisson Mixture model (30) to estimate time since infection for specimens that were collected within 6 months post infection. This model provides estimates for the number of founder viruses and time since infection based on the intersequence Hamming distribution of envelope gene sequences. In 22 of 25 specimens, the model estimates were found to be consistent with independent estimates of infection time based on HIV RNA test dates and/or seroconversion dates. This high level of consistency suggested that our UMI labeling and consensus sequence approach provided a high level of accuracy in quantifying nucleotide base differences among circulating HIV strains within an infected individual.

The limitations of the present study included the variability in the number of full-length envelope gene sequences obtained from individual specimens, which ranged from 5 to 62. A limited number of sequences can make it challenging to detect similar sequences, especially in cases of a high number of multiple founder infections. Additionally, the lowest recorded viral load for the specimens we have processed was 1,230 copies/mL, implying potential difficulties in processing specimens with low viral loads using HIV microdrop sequencing. Further refinement of the workflow might help address these challenges. The restricted availability of high-throughput sequencing is also one of the limitations of our proposed incidence surveillance, particularly in low- and middle-income countries. To address this, global sequencing core services and low-cost open-source laboratory automation can be utilized (36). It is vital to prioritize increasing access to genomics in low- and middle-income countries, as advised by the World Health Organization (WHO) (37), as this strategic support will enhance the effectiveness of our surveillance approach.

In summary, we demonstrated that the incidence assay’s metric can be determined with high precision in a high-throughput sequencing setting, which is instrumental for high-precision incidence surveillance on a large scale. Additionally, our modeling estimated the distribution of genome similarity index over time, enabling us to assess the accuracy of identifying recently infected individuals. Our high-resolution approach has the potential to maximize the utility of HIV incidence screening for case-based surveillance in public health efforts.

## Data Availability

The envelope gene sequences reported in this manuscript are available in the GenBank database (accession numbers: OP976386 - OP977462 and OP977785 - OP977930).

## SUPPLEMENTAL MATERIAL

Supplemental data and figures spectrum.02285-23-s0001.pdf Supplemental materials.

ASM does not own the copyrights to Supplemental Material that may be linked to, or accessed through, an article. The authors have granted ASM a non-exclusive, world-wide license to publish the Supplemental Material files. Please contact the corresponding author directly for reuse.
