# Supplementary material for: Precision detection of recent HIV infections using high-throughput genomic incidence assay
Source: Microbiol Spectr. 2023 Sep 15;11(5):e02285-23. doi: 10.1128/spectrum.02285-23 (PMC10580985; doi:10.1128/spectrum.02285-23)
Supplement: Supplemental data and figures — Supplemental materials. [file spectrum.02285-23-s0001.pdf]

Supplementary Material

Precision detection of recent HIV infections using high-throughput genomic incidence assay

Gina Faraci[1], Sung Yong Park[1], Tanzy M. T. Love[2], Michael P. Dubé[3], and Ha Youn Lee[1*]

[1]Department of Molecular Microbiology and Immunology, Keck School of Medicine, University of Southern California, Los Angeles, CA, Unites States.
[2]Department of Biostatistics and Computational Biology, School of Medicine and Dentistry, University of Rochester, Rochester, NY, Unites States.
[3]Department of Medicine and Division of Infectious Diseases, Keck School of Medicine, University of Southern California, Los Angeles, CA, United States.

Infection time estimates by HIV RNA test dates and seroconversion dates

The first sample of SC4 was collected on December 8th, 2009. This individual's HIV RNA last negative and first positive dates were March 14th, 2008, and November 17th, 2008, respectively. Therefore, we estimated that SC4's first sample was collected between 21 and 269 days after infection (Table 2). At the time of the first RNA positive date, this study participant was seronegative and thus at Fiebig stage I or II. By adding the estimated duration of Fiebig stage I or II (19.5 [13-34] days) to the elapsed time between the RNA first positive date and the date of specimen collection (21 days) [1, 2], we obtained an estimated infection duration of 40.5 [34 - 55] days for the first sample. This Fiebig staging estimate was within the interval obtained from HIV RNA test dates. Throughout the course of more than a year, eight additional samples were collected from this study participant (Table 2). The time since infection for each subsequent sample was estimated by adding the sample collection interval to the first sample's estimate.

Other study participants, SC8, SC15, SC18, SC19, SC20, SC22, SC23, SC24, and SC25 were also seronegative when the first sample was collected and the time since infection was estimated using the Fiebig estimate (Table 1). The first sample collected from study participant SC5 was seropositive, but it was estimated that this sample was taken within 77 days post infection based on the individual's HIV RNA negative test date. A middle time point of 38.5 days was used as the estimate for the time of infection. The HIV RNA negative and positive test dates for study participant SC21 indicated that the first seropositive sample was collected within 609 days since infection (Table 1). Instead of the middle point estimate, the shifted Poisson mixture model [3] was used to estimate time since infection, as detailed below.

Sources for publicly available incident and chronic specimens

We collected publicly available HIV complete envelope gene sequences as previously described [4, 5]. A total of 417 incident specimens were used to estimate GSI distribution over time, which comprised of 252 incident specimens at Fiebig stages I, II, III, IV, and V [2, 6-18] and 165 incident longitudinal specimens obtained from 43 individuals [2, 6, 7, 9, 11, 16, 19-21]. An additional 107 publicly available incident specimens were used to measure the detection accuracy of recent infections [22]. A total of 162 publicly available chronic specimens with an infection time longer than one year were analyzed to determine the false recency rate (FRR) [2, 17, 19, 23-42].

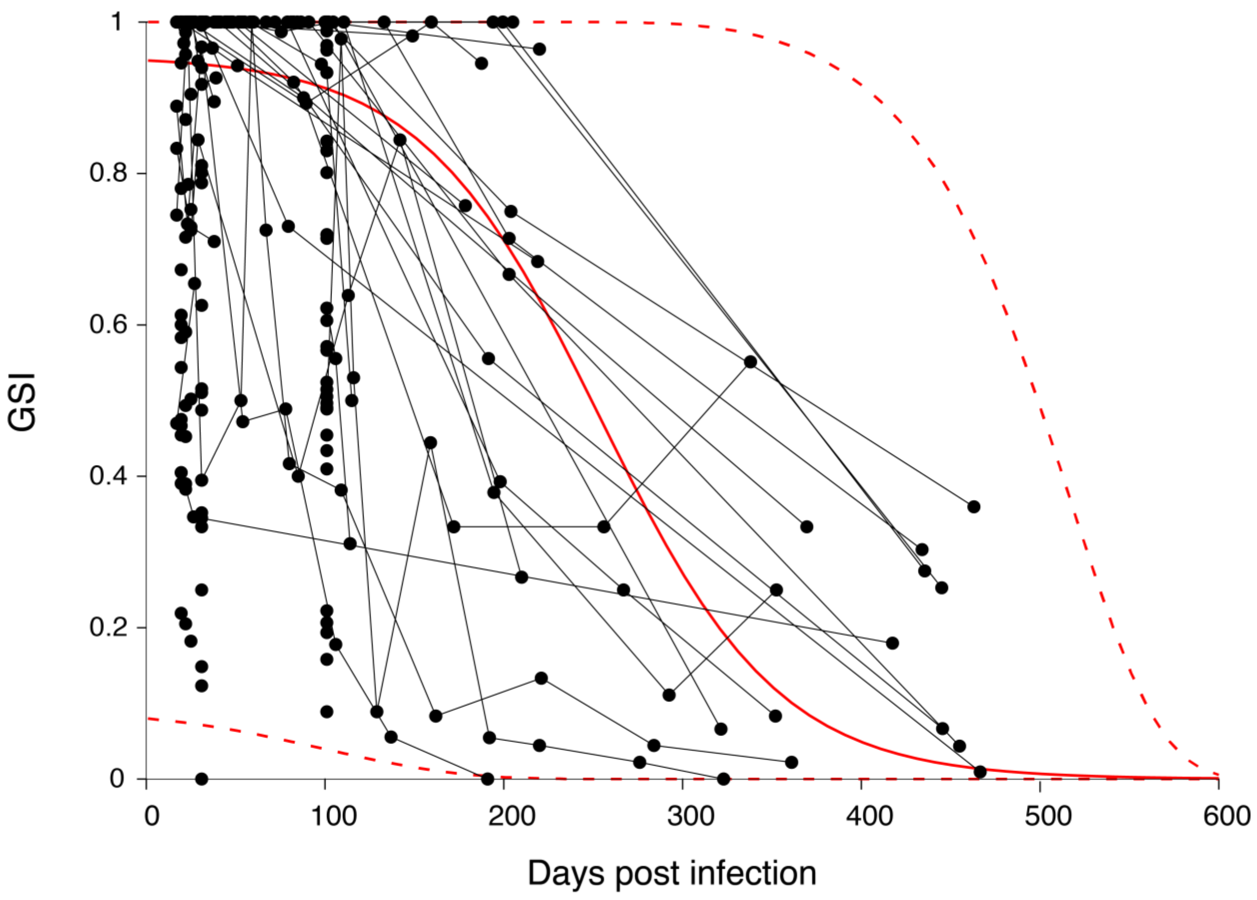

Supplementary Figure 1. Modeling biomarker dynamics. A total of 417 publicly available incident samples with infection times estimated by Fiebig staging and sample collection intervals [16, 17]. Data points with line segments represent serial samples collected from individuals. The maximum likelihood estimates of the model parameters were as follows: c = 0.95 [0.94 – 0.96], M = 253.8 [220.2 – 292.2], S = 50.1 [37.9 – 67.9], and V = 1.00 [0.94 – 1.05]. To obtain the 95% confidence interval (CI) for each parameter, we resampled the 417 incident specimens with replacement 1,000 times. The fitted mean for GSI dynamics was presented as a red solid line and 99% prediction intervals were presented as red dotted lines over days post infection.

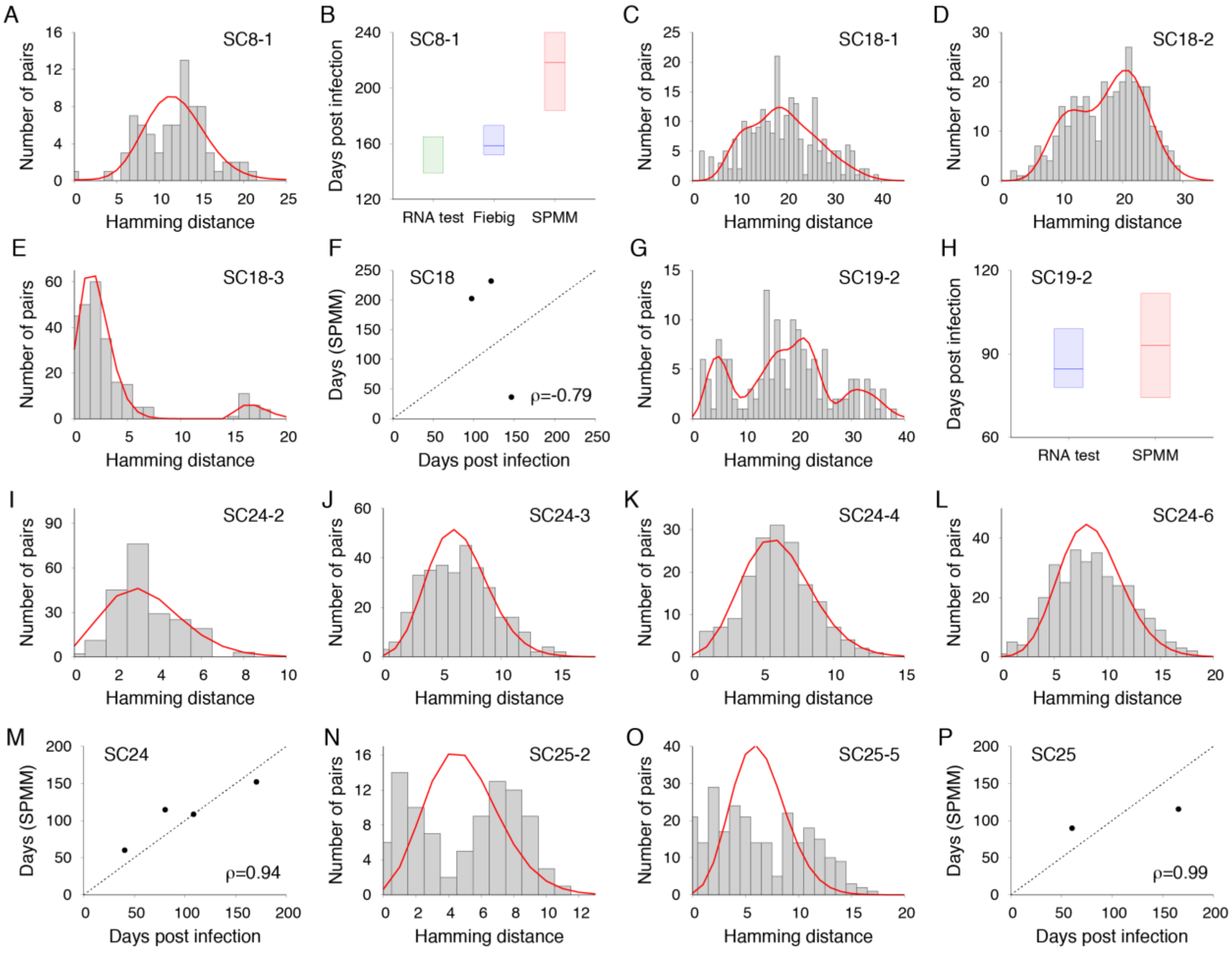

Supplementary Figure 2. Time since infection estimated by shifted Poisson mixture model (SPMM). A. The fit of SPMM (red line) to the Hamming distance distribution of SC8-1's 13 envelope gene sequences (grey boxes). **B.** Time since infection estimated by SPMM, 218.2 [183.9 – 252.5], was greater than HIV RNA test date estimate of [139 – 165] and Fiebig staging estimate of 158.5 [152 – 173] days. **C.** The fit of SPMM to the Hamming distance distribution of SC18-1. **D.** The fit of SPMM to the Hamming distance distribution of SC18-2. **E.** The fit of SPMM to the Hamming distance distribution of SC18-3. **F.** The model estimates were not consistent with HIV RNA test date estimates and Fiebig staging (Pearson correlation coefficient ρ= –0.79). **G.** The fit of SPMM to the Hamming distance distribution of SC19-2. **H.** The model estimate agreed with the infection time range determined by dates of the last negative and first positive HIV RNA tests. **I.** The fit of SPMM to the Hamming distance distribution of SC24-2. **J.** The fit of SPMM to the Hamming distance distribution of SC24-3. **K.** The fit of SPMM to the Hamming distance distribution of SC24-4. **L.** The fit of SPMM to the Hamming distance distribution of SC24-6. **M.** SPMM's infection time estimates were consistent with Fiebig estimates for the SC24's four samples (ρ=0.94). **N.** The fit of SPMM to the Hamming distance distribution of SC25-2. **O.** The fit of SPMM to the Hamming distance distribution of SC25-5. **P.** The model estimates were consistent with Fiebig estimates (ρ=0.99).

References

1. Fiebig EW, Wright DJ, Rawal BD, Garrett PE, Schumacher RT, Peddada L, et al. Dynamics of HIV viremia and antibody seroconversion in plasma donors: implications for diagnosis and staging of primary HIV infection. AIDS. 2003;17(13):1871-9.
2. Keele BF, Giorgi EE, Salazar-Gonzalez JF, Decker JM, Pham KT, Salazar MG, et al. Identification and characterization of transmitted and early founder virus envelopes in primary HIV-1 infection. Proc Natl Acad Sci U S A. 2008;105(21):7552-7.
3. Love TM, Park SY, Giorgi EE, Mack WJ, Perelson AS, Lee HY. SPMM: estimating infection duration of multivariant HIV-1 infections. Bioinformatics. 2016;32(9):1308-15.
4. Park SY, Love TM, Reynell L, Yu C, Kang TM, Anastos K, et al. The HIV Genomic Incidence Assay Meets False Recency Rate and Mean Duration of Recency Infection Performance Standards. Scientific Reports. 2017;7(1):7480.
5. Park SY, Love TMT, Kapoor S, Lee HY. HIITE: HIV-1 incidence and infection time estimator. Bioinformatics. 2018;34(12):2046-52.
6. Baalwa J, Wang S, Parrish NF, Decker JM, Keele BF, Learn GH, et al. Molecular identification, cloning and characterization of transmitted/founder HIV-1 subtype A, D and A/D infectious molecular clones. Virology. 2013;436(1):33-48.
7. Yue L, Pfafferott KJ, Baalwa J, Conrod K, Dong CC, Chui C, et al. Transmitted virus fitness and host T cell responses collectively define divergent infection outcomes in two HIV-1 recipients. PLoS Pathog. 2015;11(1):e1004565.
8. Chen Y, Li N, Zhang T, Huang X, Cai F, Vandergrift N, et al. Comprehensive Characterization of the Transmitted/Founder env Genes From a Single MSM Cohort in China. J Acquir Immune Defic Syndr. 2015;69(4):403-12.
9. Masharsky AE, Dukhovlinova EN, Verevochkin SV, Toussova OV, Skochilov RV, Anderson JA, et al. A substantial transmission bottleneck among newly and recently HIV-1-infected injection drug users in St Petersburg, Russia. J Infect Dis. 2010;201(11):1697-702.
10. Manak M, Sina S, Anekella B, Hewlett I, Sanders-Buell E, Ragupathy V, et al. Pilot studies for development of an HIV subtype panel for surveillance of global diversity. AIDS Res Hum Retroviruses. 2012;28(6):594-606.
11. Abrahams MR, Anderson JA, Giorgi EE, Seoighe C, Mlisana K, Ping LH, et al. Quantitating the multiplicity of infection with human immunodeficiency virus type 1 subtype C reveals a non-poisson distribution of transmitted variants. J Virol. 2009;83(8):3556-67.
12. Bar KJ, Li H, Chamberland A, Tremblay C, Routy JP, Grayson T, et al. Wide variation in the multiplicity of HIV-1 infection among injection drug users. J Virol. 2010;84(12):6241-7.
13. Heipertz RA, Jr., Sanders-Buell E, Kijak G, Howell S, Lazzaro M, Jagodzinski LL, et al. Molecular epidemiology of early and acute HIV type 1 infections in the United States Navy and Marine Corps, 2005-2010. AIDS Res Hum Retroviruses. 2013;29(10):1310-20.
14. Li H, Bar KJ, Wang S, Decker JM, Chen Y, Sun C, et al. High multiplicity infection by HIV-1 in men who have sex with men. PLoS Pathog. 2010;6(5):e1000890.
15. Parrish NF, Wilen CB, Banks LB, Iyer SS, Pfaff JM, Salazar-Gonzalez JF, et al. Transmitted/founder and chronic subtype C HIV-1 use CD4 and CCR5 receptors with equal efficiency and are not inhibited by blocking the integrin alpha4beta7. PLoS Pathog. 2012;8(5):e1002686.
16. Parrish NF, Gao F, Li H, Giorgi EE, Barbian HJ, Parrish EH, et al. Phenotypic properties of transmitted founder HIV-1. Proc Natl Acad Sci U S A. 2013;110(17):6626-33.
17. Gnanakaran S, Bhattacharya T, Daniels M, Keele BF, Hraber PT, Lapedes AS, et al. Recurrent signature patterns in HIV-1 B clade envelope glycoproteins associated with either early or chronic infections. PLoS Pathog. 2011;7(9):e1002209.
18. Nofemela A, Bandawe G, Thebus R, Marais J, Wood N, Hoffmann O, et al. Defining the human immunodeficiency virus type 1 transmission genetic bottleneck in a region with multiple circulating subtypes and recombinant forms. Virology. 2011;415(2):107-13.
19. Herbeck JT, Rolland M, Liu Y, McLaughlin S, McNevin J, Zhao H, et al. Demographic processes affect HIV-1 evolution in primary infection before the onset of selective processes. J Virol. 2011;85(15):7523-34.
20. Liu Y, McNevin J, Cao J, Zhao H, Genowati I, Wong K, et al. Selection on the human immunodeficiency virus type 1 proteome following primary infection. J Virol. 2006;80(19):9519-29.

21. Salazar-Gonzalez JF, Salazar MG, Keele BF, Learn GH, Giorgi EE, Li H, et al. Genetic identity, biological phenotype, and evolutionary pathways of transmitted/founder viruses in acute and early HIV-1 infection. J Exp Med. 2009;206(6):1273-89.
22. Rolland M, Edlefsen PT, Larsen BB, Tovanabutra S, Sanders-Buell E, Hertz T, et al. Increased HIV-1 vaccine efficacy against viruses with genetic signatures in Env V2. Nature. 2012;490(7420):417-20.
23. Bailey JR, Lassen KG, Yang HC, Quinn TC, Ray SC, Blankson JN, et al. Neutralizing antibodies do not mediate suppression of human immunodeficiency virus type 1 in elite suppressors or selection of plasma virus variants in patients on highly active antiretroviral therapy. J Virol. 2006;80(10):4758-70.
24. Yoshida I, Sugiura W, Shibata J, Ren F, Yang Z, Tanaka H. Change of positive selection pressure on HIV-1 envelope gene inferred by early and recent samples. PLoS One. 2011;6(4):e18630.
25. Ren C, Liu S, Li Y, Zhuang M, Yu H, Wang J, et al. Cross-neutralizing antibody profile of Chinese HIV-1-infected individuals and the viral envelope features from elite neutralizers. J Acquir Immune Defic Syndr. 2014;67(5):472-80.
26. Rong R, Li B, Lynch RM, Haaland RE, Murphy MK, Mulenga J, et al. Escape from autologous neutralizing antibodies in acute/early subtype C HIV-1 infection requires multiple pathways. PLoS Pathog. 2009;5(9):e1000594.
27. Bunnik EM, Pisas L, van Nuenen AC, Schuitemaker H. Autologous neutralizing humoral immunity and evolution of the viral envelope in the course of subtype B human immunodeficiency virus type 1 infection. J Virol. 2008;82(16):7932-41.
28. van Gils MJ, Bunnik EM, Burger JA, Jacob Y, Schweighardt B, Wrin T, et al. Rapid escape from preserved cross-reactive neutralizing humoral immunity without loss of viral fitness in HIV-1-infected progressors and long-term nonprogressors. J Virol. 2010;84(7):3576-85.
29. Daniels RS, Wilson P, Patel D, Longhurst H, Patterson S. Analysis of full-length HIV type 1 env genes indicates differences between the virus infecting T cells and dendritic cells in peripheral blood of infected patients. AIDS Res Hum Retroviruses. 2004;20(4):409-13.
30. Doria-Rose NA, Schramm CA, Gorman J, Moore PL, Bhiman JN, DeKosky BJ, et al. Developmental pathway for potent V1V2-directed HIV-neutralizing antibodies. Nature. 2014;509(7498):55-62.
31. Wu X, Wang C, O'Dell S, Li Y, Keele BF, Yang Z, et al. Selection pressure on HIV-1 envelope by broadly neutralizing antibodies to the conserved CD4-binding site. J Virol. 2012;86(10):5844-56.
32. Blish CA, Dogan OC, Derby NR, Nguyen MA, Chohan B, Richardson BA, et al. Human immunodeficiency virus type 1 superinfection occurs despite relatively robust neutralizing antibody responses. J Virol. 2008;82(24):12094-103.
33. Piantadosi A, Chohan B, Chohan V, McClelland RS, Overbaugh J. Chronic HIV-1 infection frequently fails to protect against superinfection. PLoS Pathog. 2007;3(11):e177.
34. Geels MJ, Cornelissen M, Schuitemaker H, Anderson K, Kwa D, Maas J, et al. Identification of sequential viral escape mutants associated with altered T-cell responses in a human immunodeficiency virus type 1-infected individual. J Virol. 2003;77(23):12430-40.
35. Sturdevant CB, Joseph SB, Schnell G, Price RW, Swanstrom R, Spudich S. Compartmentalized replication of R5 T cell-tropic HIV-1 in the central nervous system early in the course of infection. PLoS Pathog. 2015;11(3):e1004720.
36. Ssemwanga D, Lyagoba F, Ndembi N, Mayanja BN, Larke N, Wang S, et al. Multiple HIV-1 infections with evidence of recombination in heterosexual partnerships in a low risk Rural Clinical Cohort in Uganda. Virology. 2011;411(1):113-31.
37. Skar H, Gutenkunst RN, Wilbe Ramsay K, Alaeus A, Albert J, Leitner T. Daily sampling of an HIV-1 patient with slowly progressing disease displays persistence of multiple env subpopulations consistent with neutrality. PLoS One. 2011;6(8):e21747.
38. Ping LH, Joseph SB, Anderson JA, Abrahams MR, Salazar-Gonzalez JF, Kincer LP, et al. Comparison of viral Env proteins from acute and chronic infections with subtype C human immunodeficiency virus type 1 identifies differences in glycosylation and CCR5 utilization and suggests a new strategy for immunogen design. J Virol. 2013;87(13):7218-33.
39. Mukhopadhyay S, Ringe R, Patil A, Paranjape R, Bhattacharya J. Characterization of circulating HIV type 1 env genes in plasma of two antiretroviral-naive slow progressing patients with broad neutralizing antibody response with evidence of recombination. AIDS Res Hum Retroviruses. 2012;28(7):739-45.
40. Malherbe DC, Pissani F, Sather DN, Guo B, Pandey S, Sutton WF, et al. Envelope variants circulating as initial neutralization breadth developed in two HIV-infected subjects stimulate multiclade neutralizing antibodies in rabbits. J Virol. 2014;88(22):12949-67.

41. Evering TH, Kamau E, St Bernard L, Farmer CB, Kong XP, Markowitz M. Single genome analysis reveals genetic characteristics of Neuroadaptation across HIV-1 envelope. Retrovirology. 2014;11:65.
42. Liao HX, Lynch R, Zhou T, Gao F, Alam SM, Boyd SD, et al. Co-evolution of a broadly neutralizing HIV-1 antibody and founder virus. Nature. 2013;496(7446):469-76.
